# Probability of sporadic lymphangioleiomyomatosis in women presenting with spontaneous pneumothorax

**DOI:** 10.1186/s13023-023-02784-5

**Published:** 2023-07-06

**Authors:** Audrey Suter, Marie-Eve Müller, Cécile Daccord, Patrick Taffé, Romain Lazor

**Affiliations:** 1 Medical School, University of Lausanne, Lausanne, Switzerland; 2 Respiratory Medicine Department, Lausanne University Hospital, University of Lausanne, Lausanne, Switzerland; 3 Division of Biostatistics, Center for Primary Care and Public Health, University of Lausanne, Lausanne, Switzerland; 4 Service de Pneumologie, Centre Hospitalier Universitaire Vaudois, BU44.07.2137, Rue du Bugnon 46, 1011 Lausanne, Switzerland

**Keywords:** Lymphangioleiomyomatosis, Spontaneous pneumothorax, Primary spontaneous pneumothorax, Prevalence, Bayes theorem, Meta-analysis

## Abstract

**Background:**

Sporadic lymphangioleiomyomatosis (S-LAM) is a rare low-grade neoplasm of young women characterized by multiple pulmonary cysts leading to progressive dyspnea and recurrent spontaneous pneumothorax (SP). The diagnosis of S-LAM may be delayed by several years. To reduce this delay, chest computed tomography (CT) screening has been proposed to uncover cystic lung disease in women presenting with SP. However, the probability to discover S-LAM in this population has not been determined precisely. The aim of this study was to calculate the probability of finding S-LAM in women presenting with (a) SP, and (b) apparent primary SP (PSP) as first manifestation of S-LAM.

**Methods:**

Calculations were made by applying the Bayes theorem to published epidemiological data on S-LAM, SP and PSP. Each term of the Bayes equation was determined by meta-analysis, and included: (1) the prevalence of S-LAM in the general female population, (2) the incidence rate of SP and PSP in the general female population, and (3) the incidence rate of SP and apparent PSP in women with S-LAM.

**Results:**

The prevalence of S-LAM in the general female population was 3.03 per million (95% confidence interval 2.48, 3.62). The incidence rate of SP in the general female population was 9.54 (8.15, 11.17) per 100,000 person-years (p-y). The incidence rate of SP in women with S-LAM was 0.13 (0.08, 0.20). By combining these data in the Bayes theorem, the probability of finding S-LAM in women presenting with SP was 0.0036 (0.0025, 0.0051). For PSP, the incidence rate in the general female population was 2.70 (1.95, 3.74) per 100,000 p-y. The incidence rate of apparent PSP in women with S-LAM was 0.041 (0.030, 0.055). With the Bayes theorem, the probability of finding S-LAM in women presenting with apparent PSP as first disease manifestation was 0.0030 (0.0020, 0.0046). The number of CT scans to perform in women to find one case of S-LAM was 279 for SP and 331 for PSP.

**Conclusion:**

The probability of discovering S-LAM at chest CT in women presenting with apparent PSP as first disease manifestation was low (0.3%). Recommending chest CT screening in this population should be reconsidered.

**Supplementary Information:**

The online version contains supplementary material available at 10.1186/s13023-023-02784-5.

## Introduction

Pulmonary lymphangioleiomyomatosis (LAM) is a rare low-grade neoplasm which exclusively affects women of reproductive age. It is characterized by infiltration of the lungs by neoplastic smooth muscle-like cells (LAM cells) leading to the development of multiple pulmonary cysts, which progressively replace the lung parenchyma [1, 2] and may lead to respiratory failure [3]. The most common initial manifestations include progressive dyspnea and multiple recurrent pneumothorax resulting from spontaneous rupture of pulmonary cysts [4]. The disease may either occur sporadically (S-LAM) or in patients with tuberous sclerosis complex (TSC-LAM), a rare genetic disorder [2].

Due to rarity of the disease, the diagnosis of S-LAM is often delayed by several years after the first symptoms [5]. As spontaneous pneumothorax (SP) is a common inaugural presentation of the disease, an earlier diagnosis could theoretically be achieved by carrying out a chest computed tomography (CT) scan after a first episode of SP to reveal multiple pulmonary cysts [5, 6]. One study suggested that a screening CT-scan in women who present with an inaugural (so-called “sentinel”) SP would allow earlier detection of patients with LAM with a favorable cost–benefit ratio [5]. In this study, it was estimated that 5–30% of non-smoking women aged 25–54 presenting with an apparently primary SP (PSP), i.e. SP occurring in the absence of known lung disease, may actually have LAM as a hidden underlying cause [5]. However, this estimate has never been assessed precisely. The probability of having LAM in a woman with apparent PSP is therefore undetermined. The goals of this study were to determine the probability of having S-LAM (a) in women presenting with SP (both primary and secondary), and (b) in women presenting with apparent PSP. Calculations were made through the Bayes theorem of conditional probability, which allows to determine the probability of an event based on prior knowledge of conditions related to this event. Each term of the Bayes equation was determined through meta-analyses of published studies, following a method previously used by our group to calculate the prevalence of Birt–Hogg–Dubé syndrome in the general population [7]. Our study was restricted to S-LAM, because the issue of apparent PSP is less relevant in TSC-LAM. Indeed, TSC frequently presents in infancy or childhood with neurological, mental or cutaneous manifestations leading to the diagnosis of this genetic disorder, and LAM is systematically looked for by chest CT-scan in women once TSC is diagnosed.

## Methods

### Overview

The classical definitions of spontaneous pneumothorax (SP), primary SP (PSP) and secondary SP (SSP) were used in this study [8–10]. SP was defined as a pneumothorax occurring in the absence of precipitating external event such as trauma or iatrogenic cause. SSP was defined as SP occurring in the context of an underlying lung disease that predisposes to SP such as emphysema, fibrosis, LAM, or other cystic lung diseases. PSP was defined as SP occurring in the absence of underlying lung disease as a predisposing factor, i.e. without detectable cause [8, 10]. *Apparent* PSP was defined as a SP occurring in the absence of known underlying lung disease, although a hidden cause is present but is undiagnosed at the time of pneumothorax occurrence, which is therefore initially considered as PSP [11, 12]. In the present study, apparent PSP in patients with S-LAM was defined as the first manifestation of S-LAM, at a time when the disease was already present but not diagnosed.

In a first set of data analyses, we calculated the probability of having S-LAM among women presenting with SP, i.e. both PSP and SSP, including in women with diagnosed S-LAM as known cause of SSP. All terms of the Bayes equation were determined by meta-analyses of published studies. They included: (1) the prevalence of S-LAM in the general female population, (2) the incidence of SP in the general female population, and (3) the incidence of SP in S-LAM.

In a second set of data analyses, we calculated the probability of having S-LAM among women presenting with apparent PSP, i.e. in women with “sentinel” pneumothorax as inaugural manifestation of S-LAM in whom the disease was not yet diagnosed, and who could therefore benefit from a screening chest CT-scan to reveal multiple lung cysts. All terms of the Bayes equation were determined by meta-analyses of published studies. They included: (1) the prevalence of S-LAM in the general female population, (2) the incidence of PSP in the general female population, and (3) the incidence of apparent PSP in S-LAM.

### Literature search

A literature search was performed in November 2021 in the PubMed, Embase, Web of Science and Cochrane Library electronic databases, and was updated in April 2023. The search was limited to full-text journal articles in English and French. Articles whose primary and secondary outcomes met the subjects of interest were selected. All articles were then reviewed to identify other studies of interest in the reference list.

To assess the incidences of SP and PSP in the general population, a search was performed with the Medical Subject Heading (MeSH) keyword “Pneumothorax/epidemiology.” To assess the probability of having S-LAM among patients with SP and apparent PSP, and the probability of experiencing SP and apparent PSP in S-LAM, a search was performed with the keywords “pneumothorax” and “lymphangioleiomyomatosis” combined with the Boolean operator “AND”. To assess the prevalence of S-LAM in the general female population, a search was performed with the keywords “lymphangioleiomyomatosis” AND (“prevalence” OR “epidemiology”).

All search strategies were conducted and reported according to the PRISMA 2020 statement [13].

### Statistics

To determine the probability of S-LAM in women presenting with SP (both primary and secondary), the Bayes formula was written as follows:

$$P({\text{S - LAM|SP}}) = \frac{{P({\text{S - LAM}}) \cdot P({\text{SP|S - LAM}})}}{{P({\text{SP}})}}$$

where $P({\text{S - LAM|SP}})$ is the probability of a woman presenting with SP to be affected by S-LAM. In the numerator of the Bayes formula, $P({\text{S - LAM}})$ is the prevalence of S-LAM in the general female population, and $P({\text{SP|S - LAM}})$ the prevalence of an SP episode in individuals suffering from S-LAM. In the denominator, $P({\text{SP}})$ is the prevalence of an SP event in the general female population.

As the two prevalences, $P({\text{SP|S - LAM}})$ and $P({\text{SP}})$, are not directly measurable, we estimated them using the following formulas [14, 15]:

$$\begin{aligned} & P({\text{SP|S - LAM}}) \cong IR_{{\text{SP|S - LAM}}} \cdot \overline{D}_{{\text{SP|S - LAM}}} \\ & P({\text{SP}}) \cong IR_{{{\text{SP}}}} \cdot \overline{D}_{{{\text{SP}}}} \\ \end{aligned}$$

where $IR_{{\text{SP|S - LAM}}}$ is the yearly incidence rate of SP in the S-LAM population and $IR_{{{\text{SP}}}}$ the yearly incidence rate of SP in the general female population, $\overline{D}_{{\text{SP|S - LAM}}}$ is the average duration of an SP episode in the S-LAM population, and $\overline{D}_{{{\text{SP}}}}$ the average duration of an SP episode in the general female population.

These formulas are valid in a steady state setting, i.e. when the total population of affected and unaffected individuals remains constant over time, and provide good approximations when the two prevalences $P({\text{SP|S - LAM}})$ and $P({\text{SP}})$ are small.

Assuming that the duration of an SP episode is similar in the S-LAM population and in the general population, i.e. $\overline{D}_{{\text{SP|S - LAM}}} = \overline{D}_{{{\text{SP}}}} = \overline{D}$, one may substitute these quantities in the Bayes formula and get:

$$\begin{aligned} P({\text{S - LAM|SP}}) & = \frac{{P({\text{S - LAM}}) \cdot P({\text{SP|S - LAM}})}}{{P({\text{SP}})}} \\ & = \frac{{{\text{Prevalence}}_{{\text{S - LAM}}} \cdot IR_{{\text{SP|S - LAM}}} \cdot \overline{D}_{{\text{SP|S - LAM}}} }}{{IR_{{{\text{SP}}}} \cdot \overline{D}_{{{\text{SP}}}} }} \\ & \cong \frac{{{\text{Prevalence}}_{{\text{S - LAM}}} \cdot IR_{{\text{SP|S - LAM}}} \cdot \overline{D}}}{{IR_{{{\text{SP}}}} \cdot \overline{D}}} \\ & = \frac{{{\text{Prevalence}}_{{\text{S - LAM}}} \cdot IR_{{\text{SP|S - LAM}}} }}{{IR_{{{\text{SP}}}} }} \\ \end{aligned}$$

The value of $\overline{D }$ was based on a recently published randomized trial on the treatment of PSP, which showed that the median time of recovery for a PSP treated conservatively was 30 days, whereas it was 16 days with interventional treatment [16].

As the incidences rates (IR) were not always reported, we additionally used the following relationship between the cumulative incidence (CI) and the incidence rate [17]:

$$\begin{aligned} IR & = \frac{E}{PT} = \frac{E}{{N \cdot \overline{T}}} \\ & = \frac{{n \cdot \overline{E}}}{{N \cdot \overline{T}}} = CI \cdot \frac{{\overline{E}}}{{\overline{T}}} \\ \end{aligned}$$

where *E* is the number of SP events and *PT* the person-time product in person-years of follow-up. When the latter was not reported, it was computed by multiplying the number *N* of individuals at risk at the beginning of the follow-up period by $\overline{T}$ the average follow-up duration.

When the cumulative incidence was reported (instead of the incidence rate), *CI* = *n*/*N*, where *n* is the number of individuals experiencing at least one SP event (i.e. one or several SP episodes), the number of SP events *E* was computed by multiplying the average number $\overline{E}$ of SP episodes per individual by the number *n* of individuals experiencing at least one SP event. In addition, when the average follow-up duration $\overline{T}$ was not reported, given the small number *E* of events in comparison to the number *N* of individuals, the person-time product $PT$ was simply computed by multiplying the number of individuals by the duration of the follow-up period (the justification comes from this formula $\overline{T} = (N - E) \cdot T/N + E/N \cdot T/2 \cong T$). When the median observation time was reported (along with the sample size and inter-quartile range or range) instead of the mean, we used the Hozo et al. formula to compute the mean [18].

As the three components in the Bayes formula were provided by different studies, a separate meta-analysis for each component was conducted. The variance of $IR$ was computed based on the Poisson distribution, and the log-transformation and delta method were applied to compute a 95% confidence interval (95%CI). For the prevalence of SP, the Freeman-Tuckey double arcsine transformation [19] was used to ensure confidence intervals covering the appropriate [0–1] support.

Also, as all studies on SP and PSP epidemiology published before July 2000 were much smaller and had smaller *IR*s than those published after July 2000, a random-effects subgroup meta-analysis was carried out with the first subgroup defined by studies published before July 2000 and the second by those published after July 2000 [20]. The same approach was used for the meta-analysis of the prevalences $P(SP)$, as they were computed based on the IRs. Finally, the pooled effect sizes estimated in each stratum (defined by publication date < July 2000, > July 2000) were used to compute the probability $P({\text{S - LAM|SP}})$ of a woman presenting with SP to be affected by S-LAM in each stratum based on Bayes formula. The multivariate delta method was used to compute the variance estimate of the logit transform of $P({\text{LAM}}|{\text{SP}})$. As SP in S-LAM is a relapsing phenomenon, relapses of SP, both in S-LAM and in the general population, where taken into account for the calculation of SP incidence. If relapses were not counted in the original publication, a correction factor was applied based on a recent meta-analysis of the relapse rate in PSP [21]. To specifically determine the relapse rate in women, we performed a meta-analysis of the articles used in this study.

As a sensitivity analysis, we repeated all calculations with an arbitrary duration of SP in S-LAM of 40 days instead of 30 days.

Once the probability of S-LAM in women presenting with SP was determined, the number needed to treat (NNT), i.e. the number of chest CT-scans to perform among women with SP to detect one case of S-LAM was calculated as follows:

$$NNT = \frac{1}{{P({\text{S - LAM|SP}}) - P({\text{S - LAM}})}}$$

where $P({\text{S - LAM|SP}})$ was computed using data published after July 2000.

To determine the probability of S-LAM in women presenting with apparent PSP, the Bayes formula was written as follows:

$$\begin{aligned} P({\text{S - LAM|PSP}}) & = \frac{{P({\text{S - LAM}}) \cdot P({\text{PSP|S - LAM}})}}{{P({\text{PSP}})}} \\ & = \frac{{{\text{Prevalence}}_{{\text{S - LAM}}} \cdot IR_{{\text{PSP|S - LAM}}} \cdot \overline{D}_{{\text{PSP|S - LAM}}} }}{{IR_{{{\text{PSP}}}} \cdot \overline{D}_{{{\text{PSP}}}} }} \\ & \cong \frac{{{\text{Prevalence}}_{{\text{S - LAM}}} \cdot IR_{{\text{PSP|S - LAM}}} \cdot \overline{D}}}{{IR_{{{\text{PSP}}}} \cdot \overline{D}}} \\ & = \frac{{{\text{Prevalence}}_{{\text{S - LAM}}} \cdot IR_{{\text{PSP|S - LAM}}} }}{{IR_{{{\text{PSP}}}} }} \\ \end{aligned}$$

The same method as described above was applied, by using data on the incidence and prevalence of PSP instead of SP in the general population, and by taking into account only inaugural episodes of SP as the first disease manifestation in the S-LAM population, at a time when the diagnosis of S-LAM was not yet established. Relapses of PSP were deliberately not included in these calculations.

## Results

### Probability of S-LAM in women with SP

#### Prevalence of S-LAM in the general female population

**Fig. 1:**
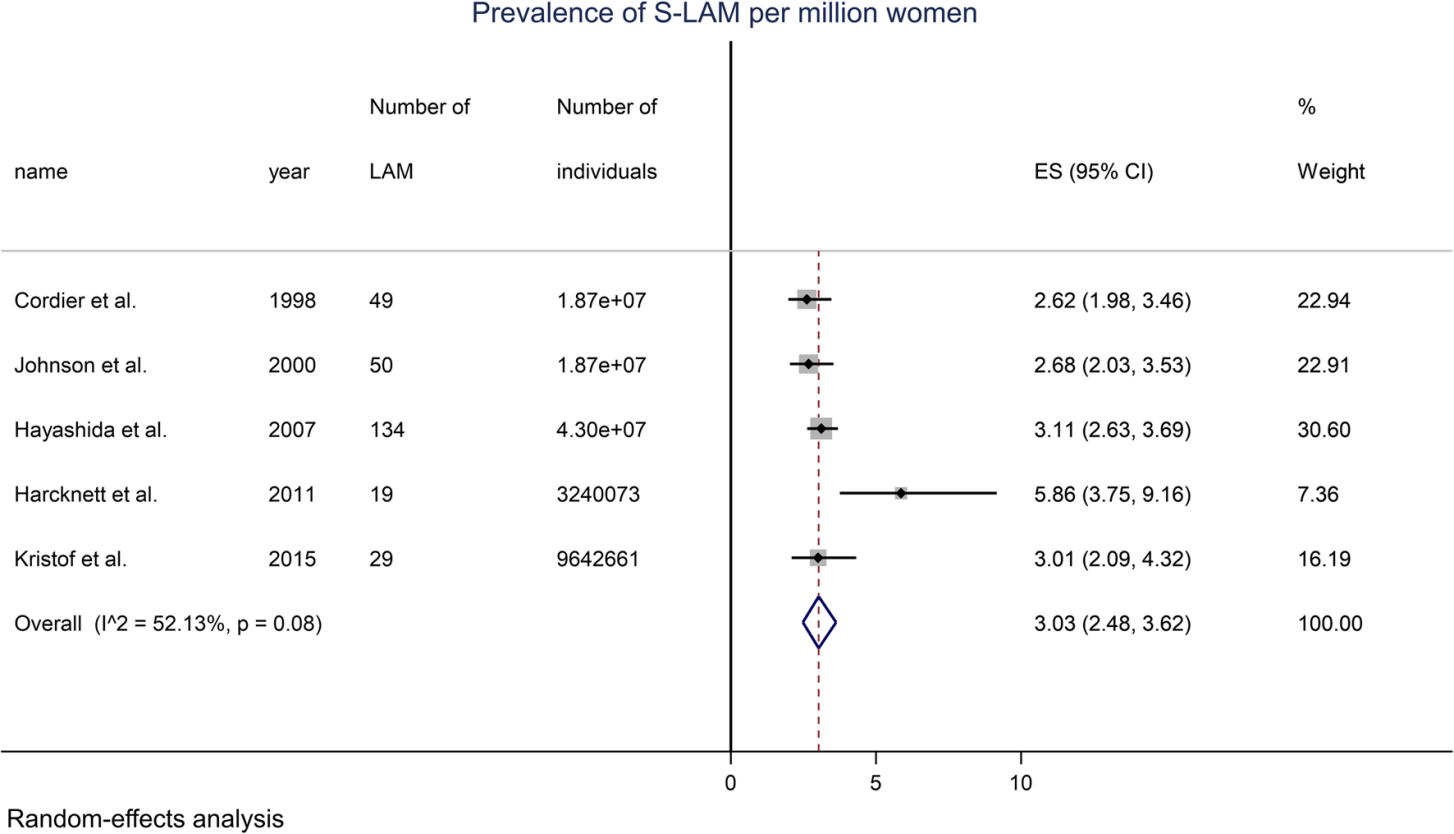
Forest plot of the prevalence of S-LAM in the general female population, random-effects model

The literature search identified 234 articles. Twenty-two original articles containing data on the prevalence of LAM were retrieved. No additional article was found after manual review. Additional file 1: Fig. S1 shows the flow diagram depicting the search strategy. Seventeen articles were excluded because the population number was missing or the TSC-LAM cases were mixed with the S-LAM cases. Thus, 5 original studies were kept for meta-analysis [4, 22–25]. Their characteristics are shown in Table 1. From reference [4] to which we have contributed with data from Switzerland, we used our own data to separate TSC-LAM and S-LAM, as this stratification was not available for other countries. By meta-analysis, the overall prevalence of S-LAM in women was 3.03 (2.48, 3.62) per million (Fig. 1).

**Table 1:** Studies reporting the prevalence of S-LAM in women

References	Country	Observation eriod	Recruited participants	Population (women)	Number of women with S-LAM
Cordier [22]	France	1991–1996	Women aged 20–69 years	18,709,718	49
Johnson [23]	UK	1994–1995	Women aged 16–65 years	18,650,000	50
Hayashida [24]	Japan	2003–2005	Women aged 20–69 years	*43,019,000*	134
Harknett [4]	Switzerland	2000–2008	Women aged 20–69 years	3,240,073	19
Kristof [25]	Québec	1996–2011	Women aged 20–69 years	*9,642,661*	29

Values in italics: results were not available in the original paper but were re-calculated from census data

#### Incidence and prevalence of SP in the general female population

The Pubmed search retrieved 1046 articles. A total of 35 original articles reporting SP incidence in the general population were retrieved. No additional article was found after manual review. Additional file 1: Fig. S2 shows the flow diagram depicting the search strategy. One paper was rejected because the number of SP could not be related to population size [26]. Sixteen articles were excluded because population size and/or gender proportion were not given. Four other articles were rejected because they focused only on PSP and not on SP. Fourteen original studies were kept for meta-analysis [27–40]. Their main characteristics are shown in Table 2.

**Table 2:** Studies reporting the incidence of spontaneous pneumothorax in women

References	Country	Observation period	Recruited participants	Patient-years (women)	Number of SP in women	Number of SP relapses in women	Number of SP in women including relapses
Hallgrimsson [27]	Iceland	1950–1974	Diagnosed with pneumothorax in any primary care setting or hospital in Iceland	*519,500*	18	3	21
Melton [28]	USA, Minnesota	1950–1974	Diagnosed with pneumothorax in any primary care setting, hospital or at autopsy in the whole county	*923,075*	30	N/A	N/A
Fergusson [29]	Scotland	1981	Diagnosed with pneumothorax at Glasgow Royal Infirmary	*1,500,000*	74	N/A	N/A
Primrose [30]	Scotland	1976–1981	Admitted for pneumothorax to one hospital respiratory unit	*630,000*	38	N/A	N/A
Bense [40]	Norway	1975–1984	Admitted consecutively in one university hospital in Oslo	578,060	35	N/A	N/A
Morales Suarez-Varela [31]	Spain	1994–1996	Diagnosed with spontaneous pneumothorax in one area of Valencia region	*340,806*	12	N/A	N/A
Gupta [32]	England and Wales	1991–1995	Diagnosed with pneumothorax in any primary care or hospital	*3,482,234*	343	N/A	N/A
Chen [33]	Taiwan	2001–2005	Admitted for spontaneous pneumothorax	*28,333,333*	1054	N/A	N/A
Bobbio [34]	France	2008–2011	Admitted for pneumothorax in any private or public hospitals in France	*124,000,000*	13,926	6713	20,639
Schnell [35]	Germany	2011–2015	Admitted for pneumothorax in any hospital in Germany AND > 10 years old	*218,000,000*	15,936	N/A	N/A
Hallifax [36]	England	2015	Admitted for pneumothorax as principal diagnosis in any public hospital AND > 15 years old	*22,978,800*	1590	237	1827
Hiyama [37]	Japan	2010–2016	Admitted for pneumothorax as main diagnosis, as recorded in a national database	*440,775,000*	27,716	N/A	N/A
Kim [38]	Korea	2002–2013	Admitted for pneumothorax in a medical service	*6,072,000*	862	N/A	N/A
Lee [39]	Korea	2014–2016	Visited emergency room for spontaneous pneumothorax, as recorded in a national database	*34,485,000*	2395	N/A	N/A

SP: spontaneous pneumothorax. Population numbers represent the yearly population multiplied by the number of years of the observation period. Values in italics: results were not available in the original paper but were re-calculated from census data. N/A: number of relapses not available in the original paper. In this case a correction factor of 0.57 was applied based on reference [21]. This correction factor (confidence interval: 0.44, 0.69) represents the relapse rate of SP in women as determined by meta-analysis of papers cited in Fig. 3 of reference [21] (data not shown)

**Fig. 2:**
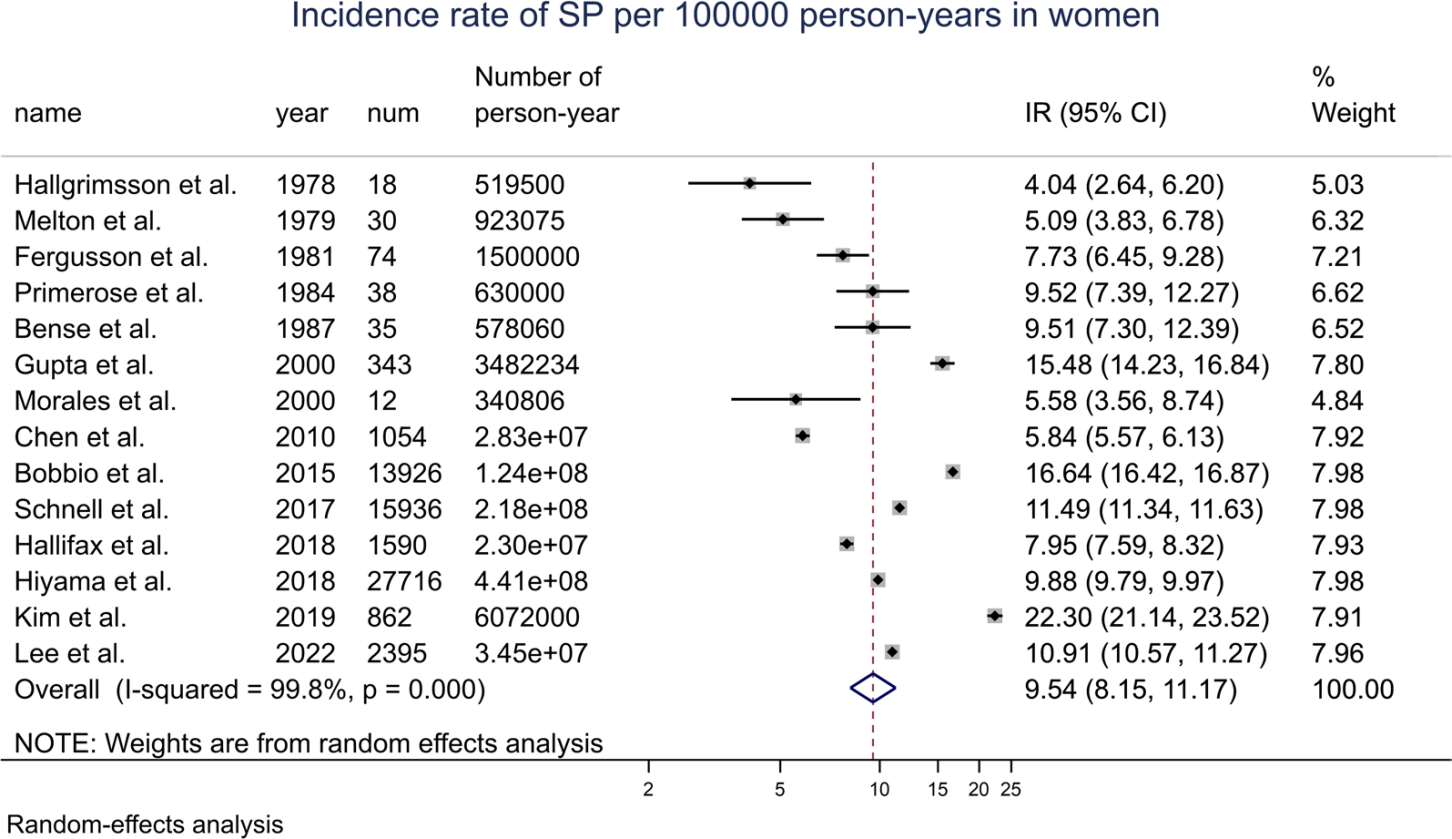
Forest plot of the incidence rate of SP for 100,000 person-years in women, random-effects model

By meta-analysis, the overall incidence rate of SP in the general female population was 9.54 (8.15, 11.17) per 100,000 p-y. With time period stratification, the incidence rate of SP in women was 6.80 (5.29, 8.76) per 100,000 p-y before July 2000, and 11.61 (9.53, 14.13) after July 2000. Figure 2 shows the results of the overall meta-analysis. Additional file 1: Fig. S3 shows the analyses by < July 2000/> July 2000 stratification.

With a random-effects model, and a 30 days SP duration, the overall prevalence of SP in the general female population was 8.40 (7.06, 9.74) per million women. It was 5.60 (4.13, 7.06) per million before July 2000, and 10.28 (8.54, 12.01) per million after July 2000. Results are detailed in Table 3 and Additional file 1: Figs. S4 and S5.

**Table 3:** Bayes equation’s components estimated by random-effects models for SP

	Value (95% confidence interval)
Prevalence of S-LAM per million women	3.03 (2.48, 3.62)
Incidence rate of SP per 100,000 p-y in the general female population, corrected for relapses	
Overall	9.54 (8.15, 11.17)
< 2000	6.80 (5.29, 8.76)
> 2000	11.61 (9.53, 14.13)
Prevalence of SP in the general female population, with SP duration of 30 days, per million women	
Overall	8.40 (7.06, 9.74)
< 2000	5.60 (4.13, 7.06)
> 2000	10.28 (8.54, 12.01)
Incidence rate of SP in women with S-LAM	0.13 (0.08, 0.20)
Prevalence of SP in women with S-LAM, with SP duration of 30 days	0.012 (0.008, 0.016)
Prevalence of S-LAM in SP, with SP duration of 30 days	
Overall	0.0044 (0.0029, 0.0066)
< 2000	0.0065 (0.0025, 0.0166)
> 2000	0.0036 (0.0025, 0.0051)
Prevalence of SP in women with S-LAM, with SP duration of 40 days	0.016 (0.011, 0.021)
Prevalence of S-LAM in SP, with SP duration of 40 days	
Overall	0.0059 (0.0039, 0.0088)
< 2000	0.0087 (0.0034, 0.0222)
> 2000	0.0048 (0.0033, 0.0069)

*SP* spontaneous pneumothorax, < 2000: before July 2000, > 2000: after July 2000

#### Incidence and prevalence of SP in women with S-LAM

The search identified 341 articles. Twenty-one original articles were retrieved. Additional file 1: Fig. S6 shows the flow diagram depicting the search strategy. One article was excluded because it focused on chest CT findings. Ten articles were excluded because the TSC- and S-LAM patients were mixed, and 2 could be kept after the values were recalculated to remove TSC-LAM patients [24, 41]. Three articles did not contain the data needed for meta-analysis. Thus, 6 original studies kept for meta-analysis [23, 24, 41–44]. Their characteristics are shown in Table 4.

**Table 4:** Studies reporting the incidence of spontaneous pneumothorax in women with S-LAM

References	Country	Observation period	Recruited participants	Population (women)	Person-time (person-years)	Number of SP	Number of apparent PSP as first symptom of S-LAM
Johnson [23]	UK	1994–1995	Women diagnosed with S-LAM	49	412	83	19
Hayashida [24]	Japan	2003	Women diagnosed with S-LAM	145	*971.5*	318	62
Oprescu [41]	USA	1995–2007	Women diagnosed with S-LAM or TSC-LAM	357	*3820*	458	101
Bee [42]	UK	2011–2015	Women diagnosed with S-LAM or TSC-LAM	106	*424*	22	N/A
Gonano [43]	France, Germany, Italy, Spain, UK, Switzerland	N/A	Women diagnosed with S-LAM or TSC-LAM	145	*1459.2*	117	57
Cheng [44]	China	2017–2020	Women diagnosed with S-LAM or TSC-LAM	345	*2998*	390	85
Johnson [45]	UK	2011–2019	Women diagnosed with S-LAM or TSC-LAM	192	*1210*	N/A	38
Johnson [45]	USA	1998–2003	Women diagnosed with S-LAM or TSC-LAM	185	*722*	N/A	50

N/A: not available. Values in italics: results were not available in the original paper but were recalculated by multiplying the number of individuals at risk by the average follow-up duration

**Fig. 3:**
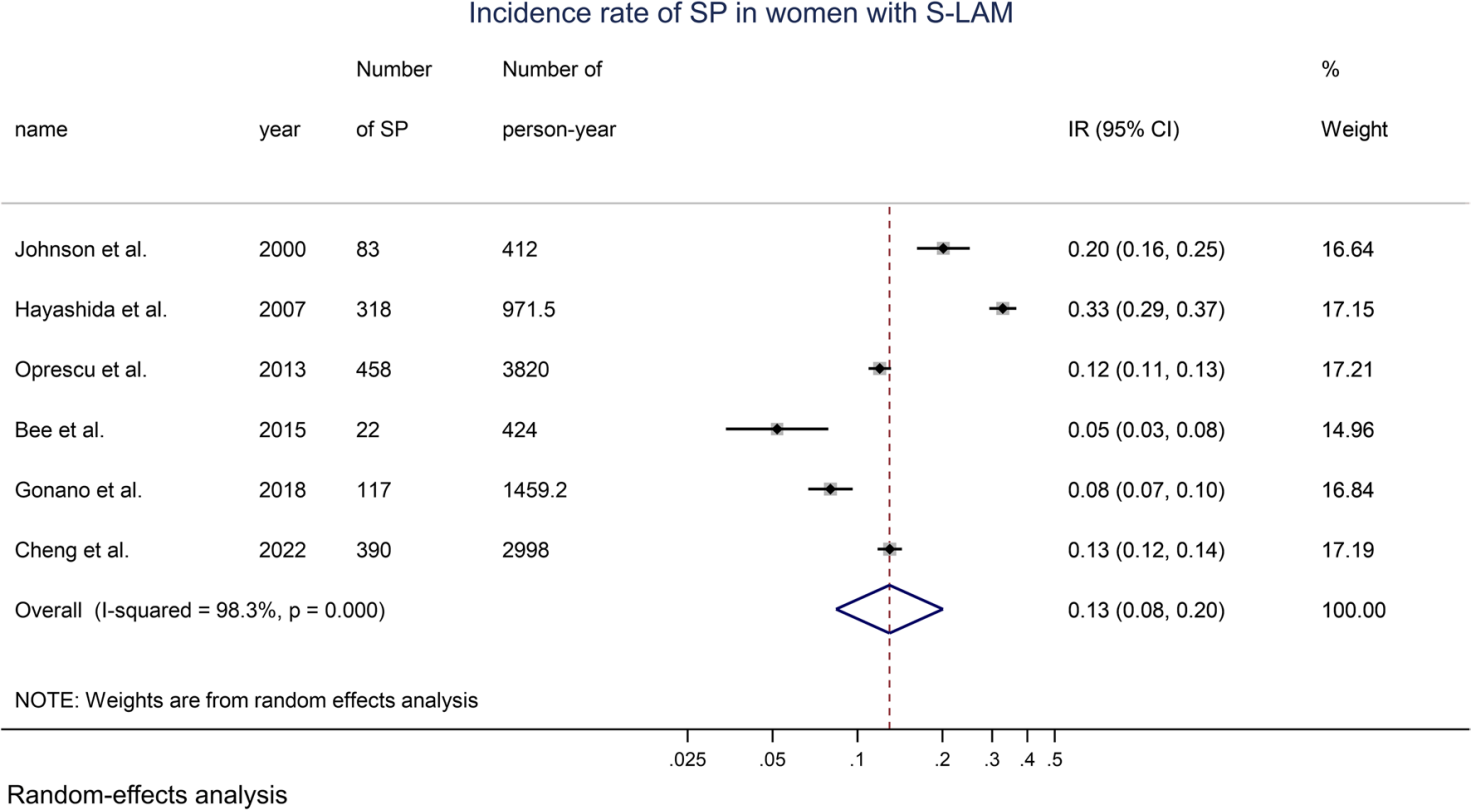
Forest plot of the incidence rate of SP in women with S-LAM, random-effects model

The annual incidence rate of SP in women with S-LAM was 0.13 (0.08, 0.20). Figure 3 shows the results of the meta-analysis. The prevalence of SP among women with S-LAM with an SP duration of 30 days was 0.012 (0.008, 0.016). Results are detailed in Additional file 1: Fig. S7.

#### Probability of S-LAM in SP

To determine the probability of finding a case of S-LAM among women presenting with SP, the above components were combined using the Bayes equation. Results are detailed in Table 3. For the calculation of SP prevalence, we assumed that the highest accuracy would be provided by studies on SP incidence published after July 2000 and by using a median pneumothorax duration of 30 days reflecting the natural history of the condition. Using these assumptions, we found a prevalence of S-LAM in SP of 0.0044 (0.0029, 0.0066). It was 0.0065 (0.0025, 0.0166) when integrating studies performed before July 2000, and 0.0036 (0.0025, 0.0051) when using studies performed after July 2000. Only slightly higher figures were found when using an arbitrary SP duration of 40 days in S-LAM instead of 30 days (Table 3). The number of CT-scans to perform among women with SP to detect one case of S-LAM was 279. As sensitivity analysis, considering the lower and upper boundaries of the confidence interval of $P({\text{S - LAM|SP}})$, this number might have varied between 195 and 400 (the uncertainty in the estimation of $P({\text{S - LAM}})$ is so small, given the large numbers, that taking it into account does not change this result).

In summary, using an SP duration of 30 days and studies on SP incidence performed after July 2000, the probability of finding S-LAM in women presenting with SP was 0.36%. The number of CT-scans to perform among women with SP to detect one case of S-LAM was 279.

### Probability of S-LAM in women with apparent PSP

#### Prevalence of S-LAM in women

This parameter of the equation was the same as the one used above to calculate the prevalence of S-LAM in women presenting with SP. The overall prevalence of S-LAM in women was 3.03 (2.48, 3.62) per million (Fig. 1).

#### Incidence and prevalence of PSP in the general population

The same literature search was conducted and 1046 original articles were identified. A total of 11 original articles reporting PSP incidence in the general population were retrieved [27, 28, 30, 34–37, 46–49]. No additional article was found after manual review. Their main characteristics are shown in Table 5. Additional file 1: Fig. S8 shows the flow diagram depicting the search strategy.

**Table 5:** Studies reporting the incidence of primary spontaneous pneumothorax in women of the general population

References	Country	Observation period	Recruited participants	Person-time in women (person-years)	Number of women with PSP
Wynn-Williams [46]	England	1947–1956	Admitted for PSP to the General hospital of a county town	*750,000*	11
Hallgrimsson [27]	Iceland	1950–1974	Diagnosed with pneumothorax in any primary care setting or hospital in Iceland	*519,500*	9
Melton [28]	USA, Minnesota	1950–1974	Diagnosed with pneumothorax in any primary care setting, hospital or at autopsy in the whole county	*923,075*	12
Primrose [30]	Scotland	1976–1981	Admitted for pneumothorax to one hospital respiratory unit	*630,000*	11
Bobbio [34]	France	2008–2011	Admitted for pneumothorax to any private or public hospital in France	*124,000,000*	12,088
Schnell [35]	Germany	2011–2015	Admitted for PSP to any hospital in Germany AND > 10 years old	*218,000,000*	12,654
Huang [47]	Taiwan	2001–2013	Admitted for PSP to a hospital in Taiwan AND > 11 and < 40 years old	*151,000,000*	*2836*
Hallifax [36]	England	2015	Admitted for pneumothorax as first diagnosis to any public hospital AND > 15 years old	*22,978,800*	564
Hiyama [37]	Japan	2010–2016	Admitted for pneumothorax as main diagnosis, as recorded in a national administrative database	*440,775,000*	18,975
Ogata [49]	Japan	2007–2013	Admitted to emergency room of one hospital for PSP	*761,905*	16
Olesen [48]	Denmark	2009–2014	Admitted for a first episode of pneumothorax to hospital AND < 40 years old	*6,818,182*	150

*PSP* primary spontaneous pneumothorax. Population numbers represents the yearly population multiplied by the number of years of the observation period. Values in italics: results were not available in the original paper but were re-calculated from census data

**Fig. 4:**
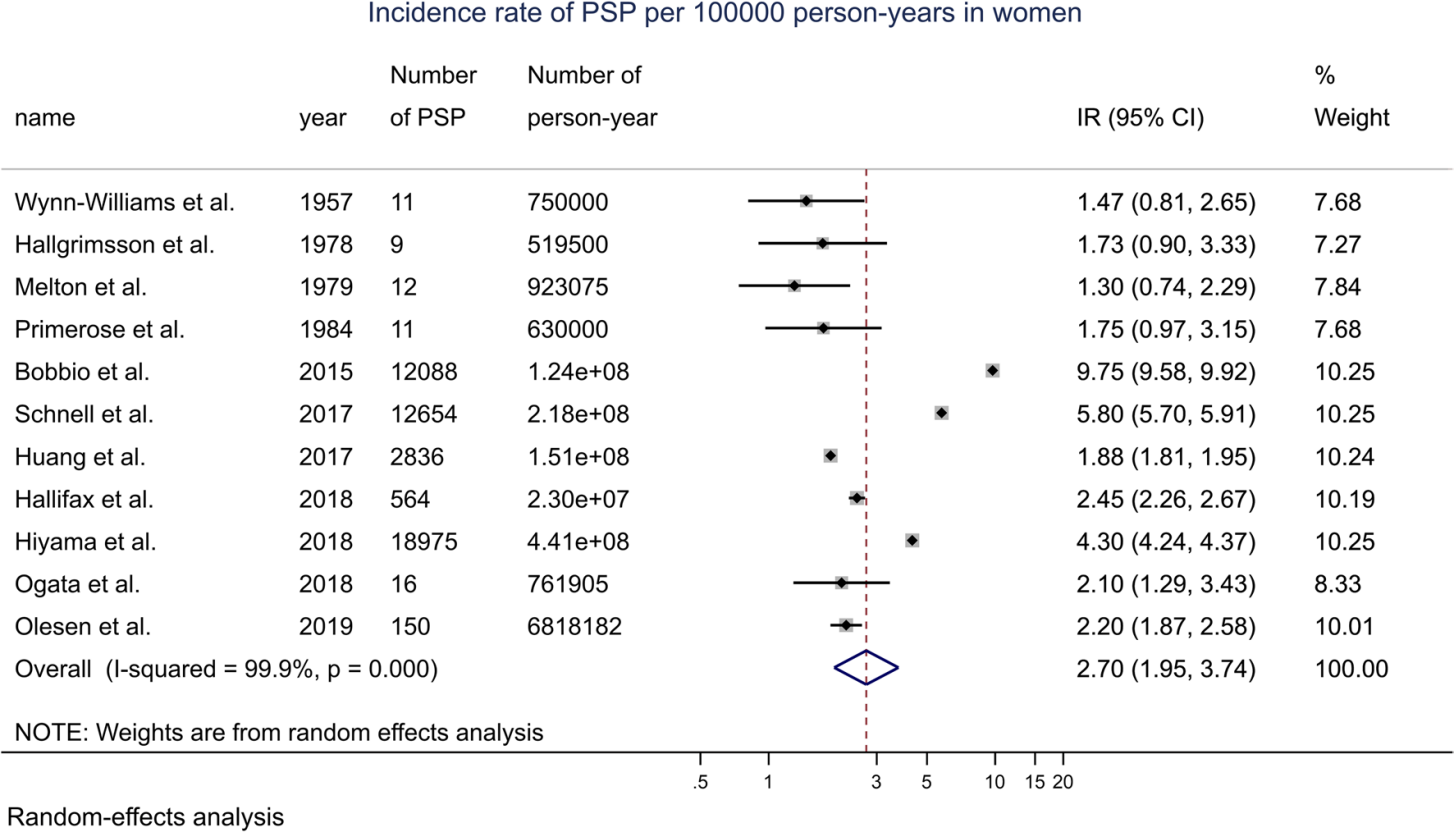
Forest plot of the incidence rate of PSP for 100,000 person-years in women, random-effects model

By meta-analysis, the overall incidence rate of PSP in the general female population was 2.70 (1.95, 3.74) per 100,000 p-y (Fig. 4). With time period stratification, the incidence rate was 1.54 (1.14, 2.07) per 100,000 p-y before July 2000, and 3.45 (2.33, 5.09) after July 2000 (Additional file 1: Fig. S9).

With a random-effects model, and a 30 days PSP duration, the overall prevalence of PSP in the general female population was 2.62 (1.46, 3.77) per million in women (Additional file 1: Fig. S10). It was 1.23 (0.86, 1.60) per million before July 2000, and 3.36 (1.92, 4.80) per million after July 2000 (Additional file 1: Fig. S11). Results are detailed in Table 6.

**Table 6:** Bayes equation’s components estimated by random-effects models for PSP

	Value (95% confidence interval)
Prevalence of S-LAM per million women	3.03 (2.48, 3.62)
Incidence rate of PSP per 100,000 p-y in the general female population	
Overall	2.70 (1.95, 3.74)
< 2000	1.54 (1.14, 2.07)
> 2000	3.45 (2.33, 5.09)
Prevalence of PSP in the general female population with PSP duration of 30 days, per million women	
Overall	2.62 (1.46, 3.77)
< 2000	1.23 (0.86, 1.60)
> 2000	3.36 (1.92, 4.80)
Annual incidence rate of apparent PSP in women with S-LAM	0.041 (0.030, 0.055)
Prevalence of apparent PSP in women with S-LAM, with PSP duration of 30 days	0.0033 (0.0026, 0.0041)
Prevalence of S-LAM in apparent PSP, with PSP duration of 30 days	
Overall	0.0038 (0.0003, 0.0066)
< 2000	0.0079 (0.0033, 0.1617)
> 2000	0.0030 (0.0020, 0.0046)
Prevalence of apparent PSP in women with S-LAM, with PSP duration of 40 days	0.0045 (0.0034, 0.0055)
Prevalence of S-LAM in apparent PSP, with PSP duration of 40 days	
Overall	0.0051 (0.0030, 0.088)
< 2000	0.0105 (0.0004, 0.2057)
> 2000	0.0040 (0.0026, 0.0061)

*PSP* primary spontaneous pneumothorax, < 2000: before July 2000, > 2000: after July 2000

#### Incidence and prevalence of apparent PSP in women with S-LAM

Seven datasets from 6 studies were identified [23, 24, 41, 43–45] and were used for the calculation of the incidence and prevalence of apparent PSP in women with S-LAM (Table 4). The number of women in whom a pneumothorax constituted the first manifestation of S-LAM at a time when the disease was undiagnosed was used as the number of apparent PSP in the study population. From reference [43] performed by our group, we reviewed our raw data to identify inaugural episodes of apparent PSP occurring before the diagnosis of S-LAM.

**Fig. 5:**
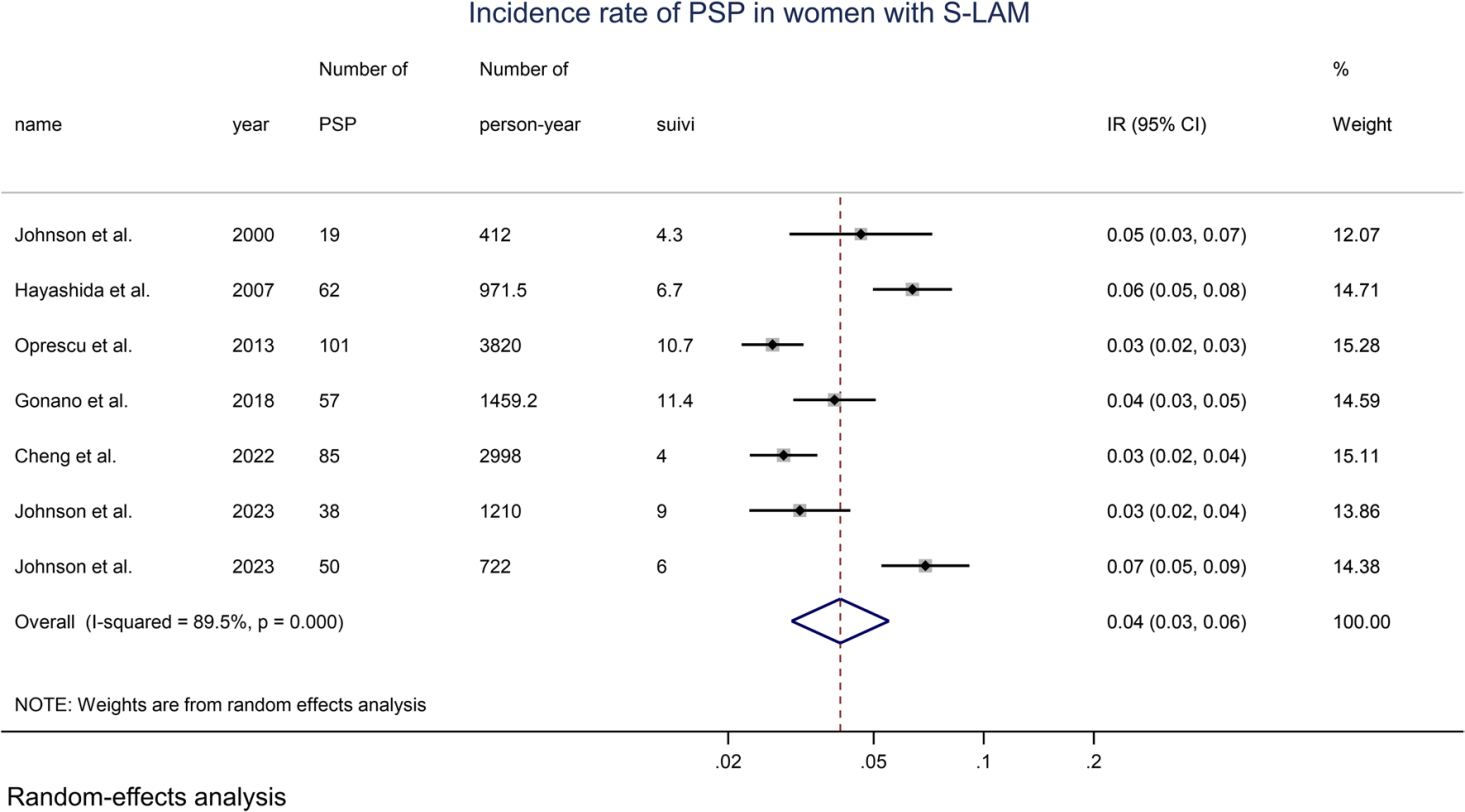
Forest plot of the incidence rate of apparent PSP in women with S-LAM, random-effects model

By meta-analysis, the annual incidence rate of apparent PSP in women with S-LAM was 0.041 (0.030, 0.055) (Fig. 5). With a PSP duration of 30 days, the overall prevalence of apparent PSP among patients with S-LAM was 0.0033 (0.0026, 0.0041) (Additional file 1: Fig. S12).

#### Probability of S-LAM in PSP

The 3 components were integrated into the Bayes equation to determine the probability of S-LAM among women presenting with apparent PSP. The results are detailed in Table 6. We found a probability of S-LAM of 0.0038 (0.0003, 0.0066) for a PSP duration of 30 days. It was 0.0079 (0.0033, 0.1617) when integrating studies before July 2000, and 0.0030 (0.0020, 0.0046) when using studies after July 2000. As sensitivity analysis, the calculation of the prevalence of S-LAM was also performed for an arbitrary PSP duration of 40 days, which led to slightly higher figures only (Table 6). The number of CT-scans to perform among women with PSP to detect one case of S-LAM was 331. As sensitivity analysis, considering the lower and upper boundaries of the confidence interval of $P({\text{S - LAM|PSP}})$, this number might have varied between 219 and 502 (the uncertainty in the estimation of $P({\text{S - LAM}})$ is so small, given the large numbers, that taking it into account does not change this result).

In summary, using a PSP duration of 30 days and studies on PSP incidence performed after July 2000, the probability of finding S-LAM in women presenting with apparent PSP was 0.3%. The number of CT-scans to perform among women with PSP to detect one case of S-LAM was 331.

## Discussion

In this study, epidemiological data on SP, PSP and S-LAM were used to calculate the probability of finding a case of S-LAM among women presenting with both primary and secondary SP, and among women presenting with apparent PSP, some of whom having in fact undiagnosed S-LAM and experiencing an inaugural episode of (sentinel) pneumothorax. Calculations were based on the Bayes theorem of conditional probability, and meta-analyses of published studies to determine each component of the Bayes equation. We found a probability of S-LAM among women presenting with SP of 0.36%, and the number of CT-scans to perform to detect one case of S-LAM was 279. The probability of S-LAM among patients presenting with apparent PSP was 0.3%, and the number of CT-scans to perform to discover one S-LAM case was 331. To our knowledge, this is the first study to precisely determine these parameters.

In a previous publication addressing this issue [5], Hagaman et al. estimated the probability of finding LAM among non-smoking women with sentinel SP, i.e. inaugural apparent PSP, to be 5–30%. Using a conservative value of 5%, these authors concluded that the NNT, i.e. the number of women with sentinel SP needed to screen to uncover on case of S-LAM, was about 20. Based on these assumptions, the cost-effectiveness of performing a systematic chest CT-scan in non-smoking women aged 25–54 presenting with inaugural apparent PSP was calculated using a Markow state-transition model. The authors concluded that this procedure was cost-effective and should be encouraged to allow earlier diagnosis of LAM. However, these conclusions relied heavily on the probability of having S-LAM among women presenting with apparent PSP, and this value has not been determined precisely, but only estimated. We calculated this parameter in the present study. Our findings sharply contrast with the results of Hagaman et al., and suggest that the probability of S-LAM among women with apparent PSP is much lower than previously expected. This leads to question the cost/benefit ratio of systematically screening all women presenting with apparent PSP by chest CT-scan. Indeed, unlike the NNT of 20 found by Hagaman et al., we found an NNT of 331, meaning that 331 women with apparent PSP need to be screened by chest CT-scan to discover one case of S-LAM. This has important implications in terms of cost-effectiveness. Hagaman et al. used a threshold of 50,000 $ per quality-adjusted life year (QALY) to define the cost-effectiveness of an intervention. In their sensitivity analysis, the lowest prevalence of LAM in the population tested was 0.8% and was associated with a cost of 85,291 $/QALY, meaning that the intervention was no longer effective at this prevalence. With the even lower probability of S-LAM in women presenting with apparent PSP found in the present study (0.3%), the intervention does not appear cost-effective. Furthermore, besides cost-effectiveness, the likelihood of help to harm should also be considered, given the number of incidental findings at chest CT-scan screening which generate additional, possibly invasive, diagnostic procedures, as shown in lung cancer screening studies [50]. Finally, the irradiation of the population exposed to chest CT-scan screening should also be considered.

The sharp contrast between the findings of Hagaman et al. and the present study has several possible explanations. First, it is unclear how the prevalence of LAM was calculated in their study. The authors cite prevalence values between 0.6 and 3 per million based on the published literature [24, 51–53]. However, in some of these references, only S-LAM was considered [51, 52], whereas others included both S-LAM and TSC-LAM [24, 53]. We chose to restrict our analysis to S-LAM, as TSC-LAM is frequently diagnosed on the basis of extra-pulmonary symptoms manifesting early in life, and the event of sentinel PSP is less relevant for the diagnosis of TSC-LAM. In addition, Hagaman et al. estimated the prevalence of LAM in the United States on the basis of the number of patients recorded in the registry of the LAM Foundation (n = 850) over a 15-year period (1995–2009). However, it is not clear whether all these cases were truly diagnosed within this period. One can hypothesize that: (1) both S-LAM and TSC-LAM were included, and (2) that, at the opening of the registry in 1995, older cases of LAM were also included, thus leading to overestimation of prevalence. The prevalence of LAM appears in the numerator of the Bayes equation. Thus, when overestimated, it contributes to overestimate the probability of LAM among women presenting with SP or PSP.

Secondly, the number of SP in this LAM population was arbitrarily estimated to 3 per patient during a 3-decades period, i.e. an incidence rate of (3 × 850)/(30 × 850) = 10% per year. This is roughly similar to the 13% found by meta-analysis in the present study. However, these SP included both SP occurring in women with known LAM (including repeated events) and inaugural apparent PSP in women with undiagnosed LAM. The latter subgroup is the true population of interest, which could theoretically benefit from a screening chest CT-scan at first apparent (sentinel) PSP. When restricting the calculation to this specific subpopulation, we found an incidence rate of first apparent PSP in LAM of only 4.1%, i.e. lower than the 10% of Hagaman et al. The incidence rate of apparent PSP in S-LAM appears in the numerator of the Bayes equation. Thus, when overestimated, it also contributes to overestimate the probability of S-LAM among women with apparent PSP.

Thirdly, based on incidence values of SP reported in the general female population between 1.2 and 9.8/100,000/year [28, 32, 40], Hagaman et al. estimated that the incidence of SP in the female population aged 25–54 was between 0.16 and 1.3/100,000/year. We found higher values by meta-analysis of recent large epidemiological studies performed after July 2000, i.e. 11.61/100,000/year for SP (Table 3) and 3.45/100,000/year for PSP (Table 6). The incidence rate of SP or PSP in the general population appears in the denominator of the Bayes equation. When underestimated, it further contributes to overestimate the probability of S-LAM among women with SP or apparent PSP. In turn, overestimating the probability of S-LAM leads to underestimate the NNT to uncover one case of LAM by chest CT-scan screening among women presenting with SP or PSP, and to overestimate the cost-effectiveness of the intervention.

Other cystic lung disease manifesting with recurrent SP such as Birt–Hogg–Dubé syndrome or pulmonary Langerhans cell histiocytosis (PLCH) could theoretically also benefit from a screening chest CT-scan at first episode of apparent PSP, and combining these diagnoses might reduce the NNT to uncover one case. However, one recent study by Cattran et al. found that LAM and PLCH taken together account for only 0.13% of SP occurring in the United States [26], which is lower than the figures found for S-LAM alone in the present study. Only hospitalized patients were considered in the study by Cattran et al. [26], which might result in underestimated figures. Additionally, it is not specified whether the SP episodes occurring in LAM and PLCH in this study were sentinel events, or whether they occurred in already diagnosed cases, in whom a screening chest CT-scan is no longer relevant.

In the present study, we used a thorough methodology previously developed by our group to determine the prevalence of Birt–Hogg–Dubé syndrome in the general population based on meta-analyses and the Bayes theorem [7]. Particular attention was paid to avoid or minimize all potential sources of bias. Studies included in meta-analyses were carefully selected using a standard methodology [13]. Mixing of S-LAM and TSC-LAM was avoided and only S-LAM was considered for the reasons mentioned above. In contrast to the study by Hagaman et al., SP and apparent PSP were considered separately in the present study. Indeed, CT-scan screening is only relevant in women presenting with apparent PSP and undiagnosed S-LAM, whereas it is of no interest in known pre-existing lung diseases, including S-LAM, presenting with recurrent SP. Separating these 2 settings is therefore essential. Although we analyzed both for clarity and completeness, only the analysis of undiagnosed S-LAM in apparent PSP is truly relevant to assess the value of CT-scan screening. For the same reasons, relapses were taken into account in the calculations made for SP, whereas for apparent PSP as sentinel event in women with undiagnosed S-LAM, relapses were deliberately not considered, and only the first event was taken into account. To determine the incidence of SP and PSP in the general population, we chose to rely on studies published after July 2000 to reflect more accurately the true incidence. Indeed, substantial differences in SP and PSP incidences were observed between studies performed before and after July 2000, the latter consistently showing a higher incidence. As a true increase in incidence over time appears unlikely, we believe that the observed differences are due to more comprehensive case finding and larger sample size in more recent studies, which were based on national registries or large medical care networks, allowing to retrieve data more precisely and at a larger scale than the small studies performed decades earlier at a regional level only (county, island, or a region smaller than a country). We thus considered that the true incidence of SP and PSP was better appraised in recent studies, and chose to rely more on data from this subgroup. The duration of PSP needed to calculate the prevalence of PSP in the general population was based on a recently published randomized controlled trial on conservative versus interventional treatment of PSP, thus allowing to determine the natural history of PSP [16]. Finally, Hagaman et al. considered only non-smoking women in their calculations, to eliminate cases of SP related to smoking. However, patients with S-LAM may also smoke, as shown in one large series where 37% of patients were active smokers or ex-smokers at the time of S-LAM diagnosis [52], a smoking prevalence similar to that of the general population. We therefore considered that women with a history of smoking should be maintained in the population at risk of having S-LAM and we did not exclude these patients from our study.

Several terms of the Bayes equation determined for the purpose of the present study deserve comments. First, to our knowledge, we provide the first determination of S-LAM prevalence by meta-analysis. Although only 5 studies were available [4, 22–25], little variation was observed between countries, suggesting that the value provided by the meta-analysis (3.03 cases per million) is close to the true disease prevalence, and that it is similar in various populations worldwide. Secondly, the annual incidence of SP in S-LAM determined by meta-analysis of 6 studies was 13%. This is higher than the 8% found in one large study by our group, which specifically addressed this issue [43]. As the 5 other studies were not specifically designed to calculate this parameter, it is possible that some bias has occurred, although data remain in the same range of magnitude. In any case, this confirms that the incidence of SP in LAM is about 1000 times higher than in the general female population.

Our study has limitations. The number of epidemiological studies on S-LAM was small. The number of studies allowing to determine the annual incidence rate SP and apparent PSP in S-LAM was also small, as was the number of patients included in each study. Thirdly, the average duration of SP in S-LAM is not known. We hypothesized that it was the same as the duration of PSP in the general population, but given the different nature of the disease, we could not rule out a longer disease course in S-LAM. To overcome this difficulty, we used pneumothorax durations of 30 and 40 days in the calculations of SP and PSP prevalence in S-LAM, and found little variability in the final probability of S-LAM among SP and apparent PSP. This reinforces the validity of our findings.

In summary, our findings question the suggestion of Hagaman et al. to perform systematic screening of women with SP or PSP by chest CT-scan in search of cases of S-LAM, and we believe that more studies are needed to explore this issue. Indeed, current guidelines on SP and PSP [8–10, 54] do not recommend systematic chest CT-scan at first episode, and suggest to perform it only in selected situations. However, our findings do not challenge to use of chest CT-scan for diagnosis and clinical management of individual patients, and it remains an invaluable tool in this setting. It is also worthwhile reminding that, for LAM as for other diseases, screening is not equivalent to diagnosis. Indeed, although multiple, round, thin-walled cysts evenly distributed throughout the lung parenchyma at chest CT-scan are highly suggestive of LAM, its diagnosis requires at least one additional feature such as increased vascular endothelial growth factor D, the presence of renal angiomyolipoma or lymphangiomas at imaging, chylous effusion, a histopathological proof of LAM, or characteristic features of TSC, in the appropriate clinical setting [55, 56].

## Conclusions

This study is the first one to precisely determine the probability of finding S-LAM among women presenting with apparent PSP. This probability determines the relevance of screening this population by systematic chest CT in search of S-LAM. We found that the probability of finding S-LAM among women with apparent PSP was only 0.3% with an NNT of 331, a very different result from that published previously [5]. This has major impact on the cost/benefit ratio and the likelihood of help to harm of this intervention. More studies are needed before recommending systematic chest CT screening in women presenting with apparent PSP in search of S-LAM and other cystic lung diseases.

## Supplementary Information

**Additional file 1. Fig S1.** PRISMA flow diagram for S-LAM epidemiology. **Fig S2.** PRISMA flow diagram for SP epidemiology. **Fig S3.** Incidence rate of SP in women stratified by period before/after July 2000. **Fig S4.** Overall prevalence of SP in women with average pneumothorax duration of 30 days. **Fig S5.** Prevalence of SP in women stratified by period before/after July 2000, with average pneumothorax duration of 30 days. **Fig S6.** PRISMA flow diagram for SP and PSP in women with S-LAM. **Fig S7.** Prevalence of SP in women with S-LAM with average duration of SP of 30 days, random-effects model. **Fig S8.** PRISMA flow diagram for PSP epidemiology. **Fig S9.** Incidence rate of PSP in women of the general population stratified by period before/after July 2000. **Fig S10.** Overall prevalence of PSP in women of the general population, with an average PSP duration of 30 days. **Fig S11.** Prevalence of PSP in women of the general population stratified by period before/after July 2000, with average PSP duration of 30 days. **Fig S12.** Prevalence of apparent PSP in women with S-LAM, with average duration of PSP of 30 days, random-effects model.

## Data Availability

Data used in this study are available from the authors upon reasonable request.

## Abbreviations

LAM: Lymphangioleiomyomatosis
p-y: Person-years
SP: Spontaneous pneumothorax
PSP: Primary spontaneous pneumothorax
P: Prevalence
IR: Incidence rate
