# Supplementary material for: Probability of sporadic lymphangioleiomyomatosis in women presenting with spontaneous pneumothorax
Source: Orphanet J Rare Dis. 2023 Jul 6;18:180. doi: 10.1186/s13023-023-02784-5 (PMC10327335; doi:10.1186/s13023-023-02784-5)

PROBABILITY OF SPORADIC LYMPHANGIOLEIOMYOMATOSIS IN WOMEN PRESENTING WITH SPONTANEOUS PNEUMOTHORAX

Supplementary material

Table of contents

Supplementary figure S1. PRISMA flow diagram for S-LAM epidemiology 2

Supplementary figure S2. PRISMA flow diagram for SP epidemiology 3

Supplementary figure S3. Incidence rate of SP in women stratified by period before/after July 2000 4

Supplementary figure S4. Overall prevalence of SP in women 5

Supplementary figure S5. Prevalence of SP in women stratified by period before/after July 2000 6

Supplementary figure S6. PRISMA flow diagram for SP and PSP in women with S-LAM..... 7

Supplementary figure S7. Prevalence of SP in women with S-LAM 8

Supplementary figure S8. PRISMA flow diagram for PSP epidemiology 9

Supplementary figure S9. Incidence rate of PSP in women of the general population stratified by period before/after July 2000 10

Supplementary figure S10. Overall prevalence of PSP in women of the general population, with an average PSP duration of 30 days 11

Supplementary figure S11. Prevalence of PSP in women of the general population, with an average PSP duration of 30 days, stratified by period before/after July 2000 12

Supplementary figure S 12. Prevalence of apparent PSP in women with S-LAM, with average duration of PSP of 30 days, random-effects model. 13

Supplementary figure S1. PRISMA flow diagram for S-LAM epidemiology

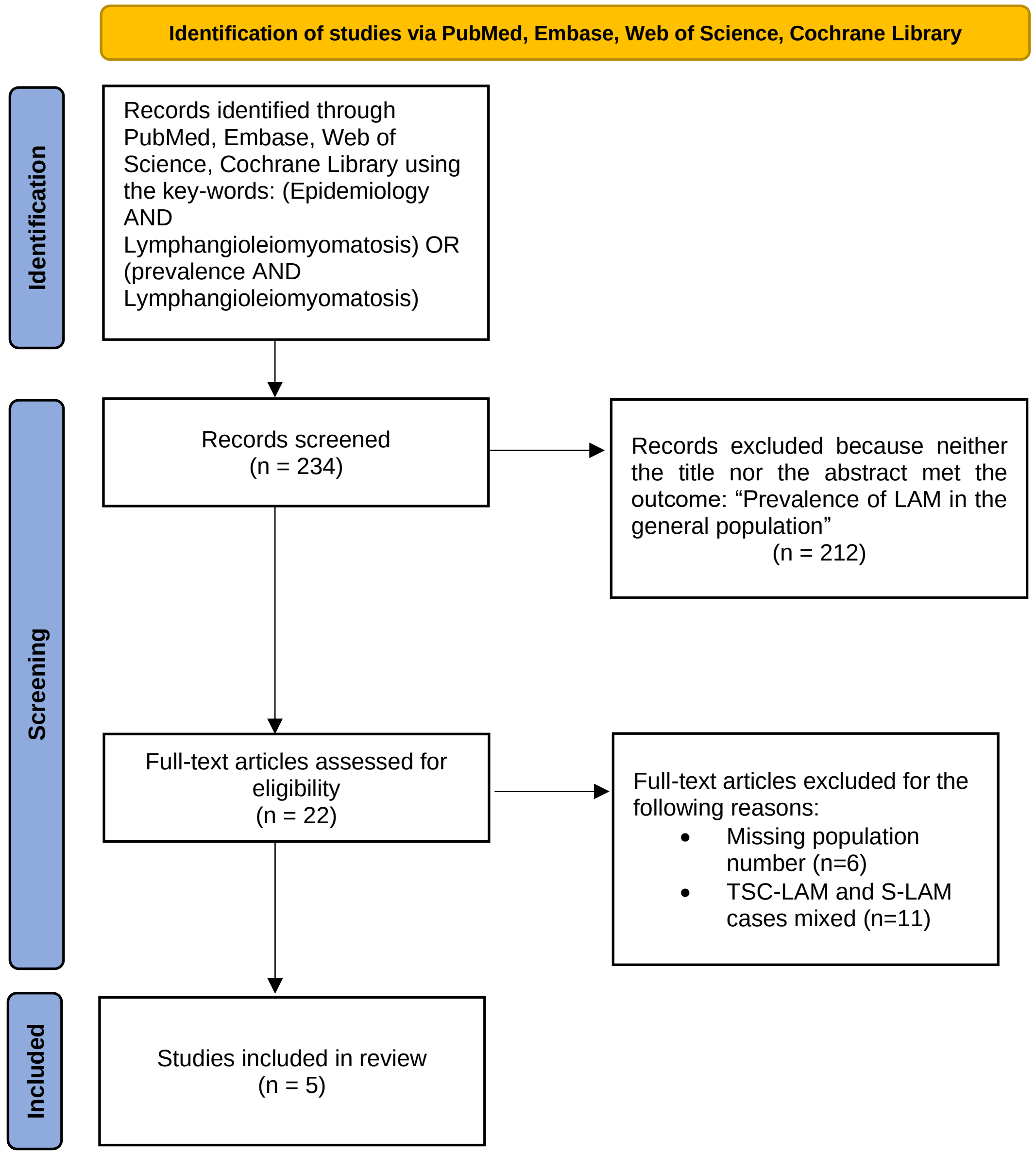

Supplementary figure S2. PRISMA flow diagram for SP epidemiology

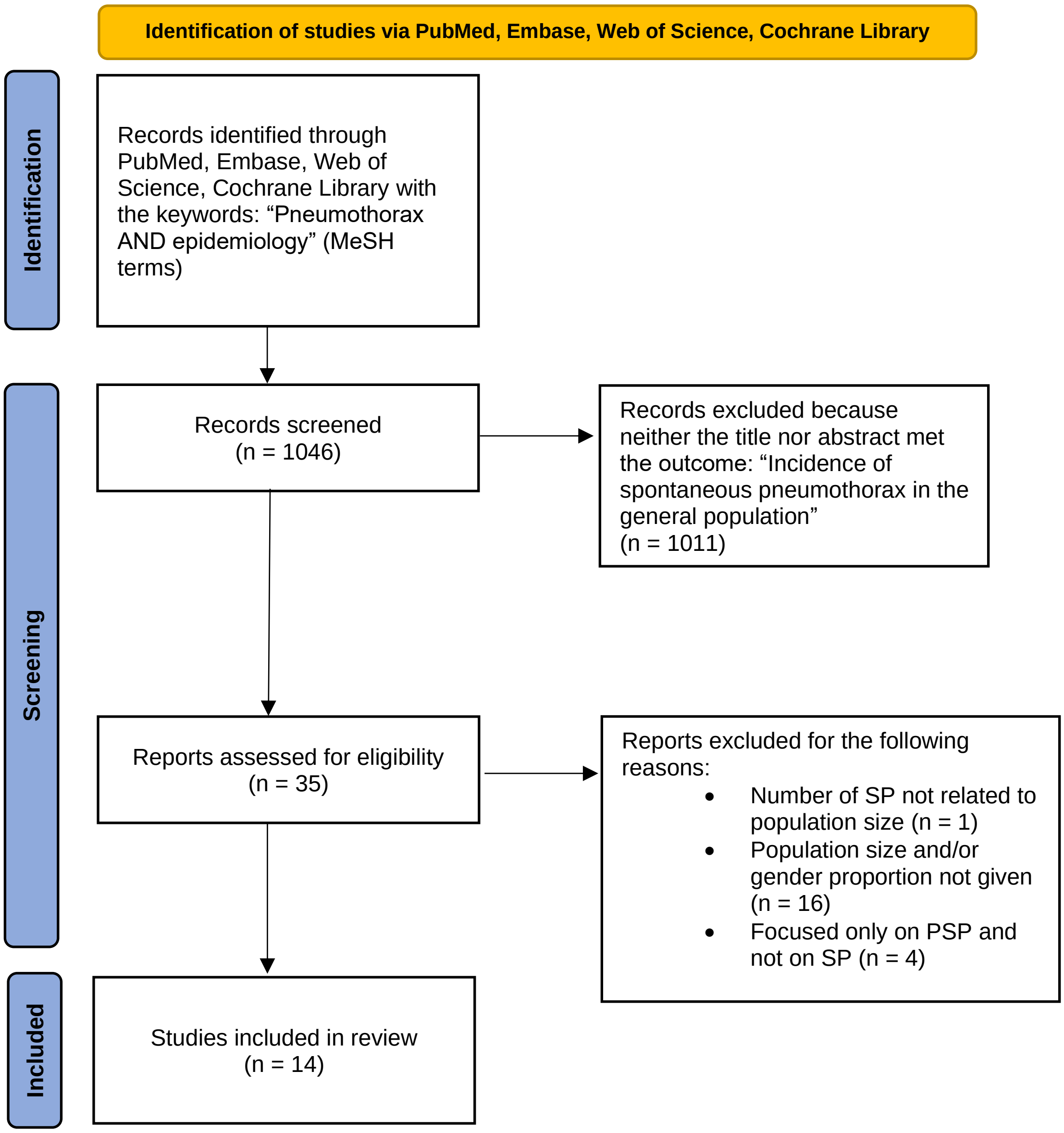

Supplementary figure S3. Incidence rate of SP in women stratified by period before/after July 2000

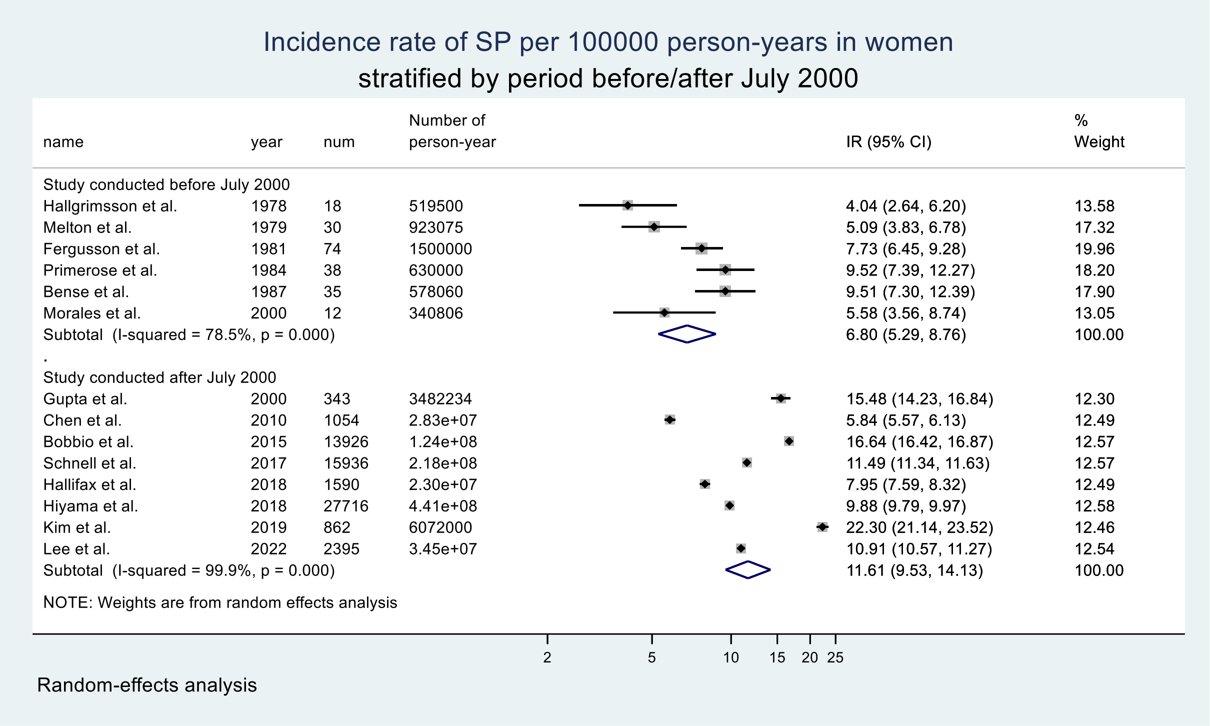

Supplementary figure S4. Overall prevalence of SP in women

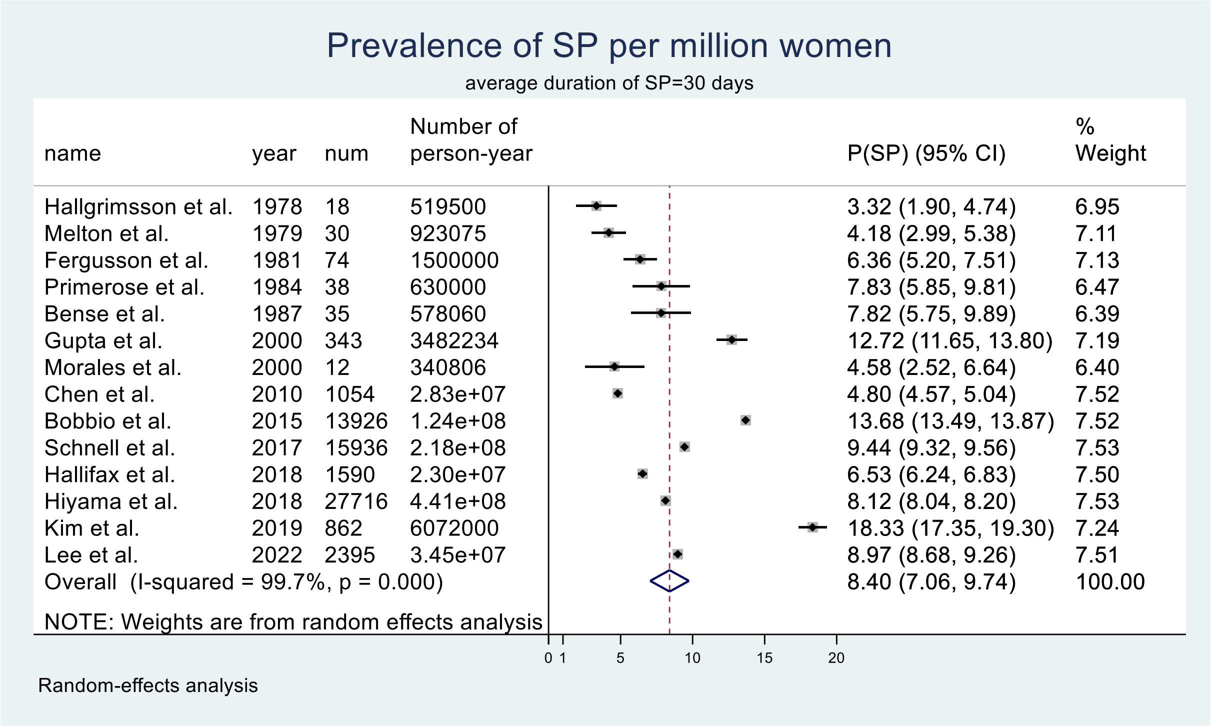

Supplementary figure S5. Prevalence of SP in women stratified by period before/after July 2000

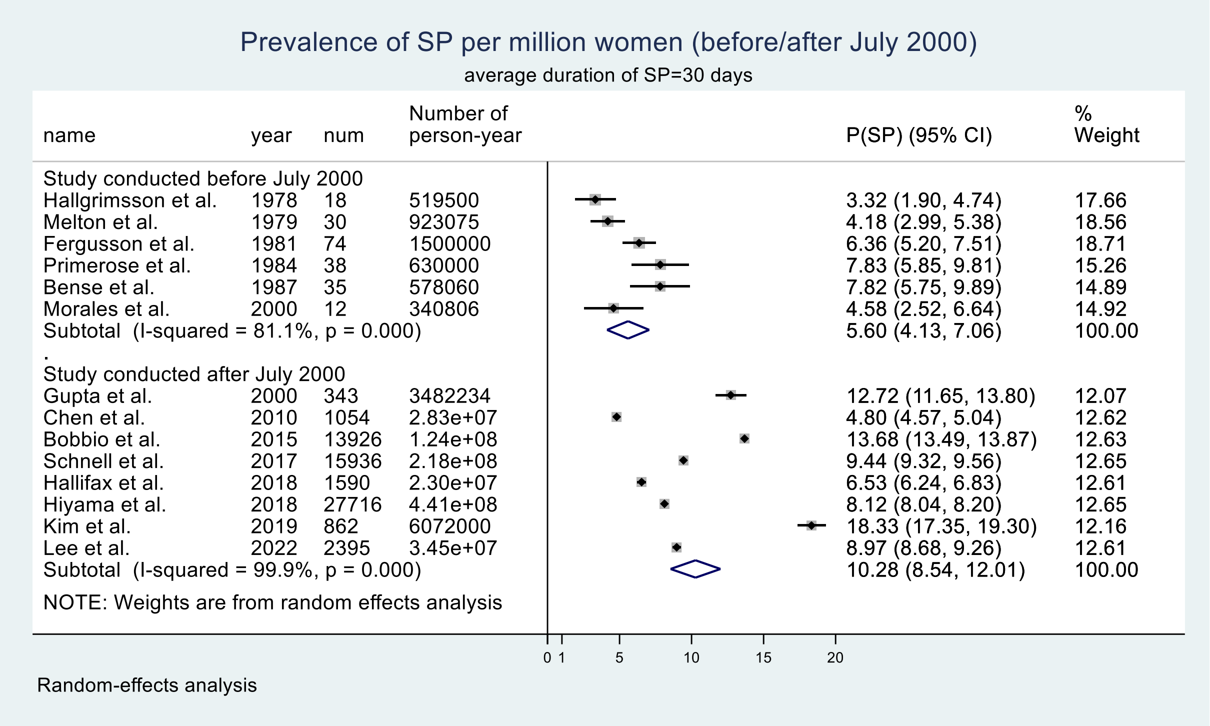

Supplementary figure S6. PRISMA flow diagram for SP and PSP in women with S-LAM

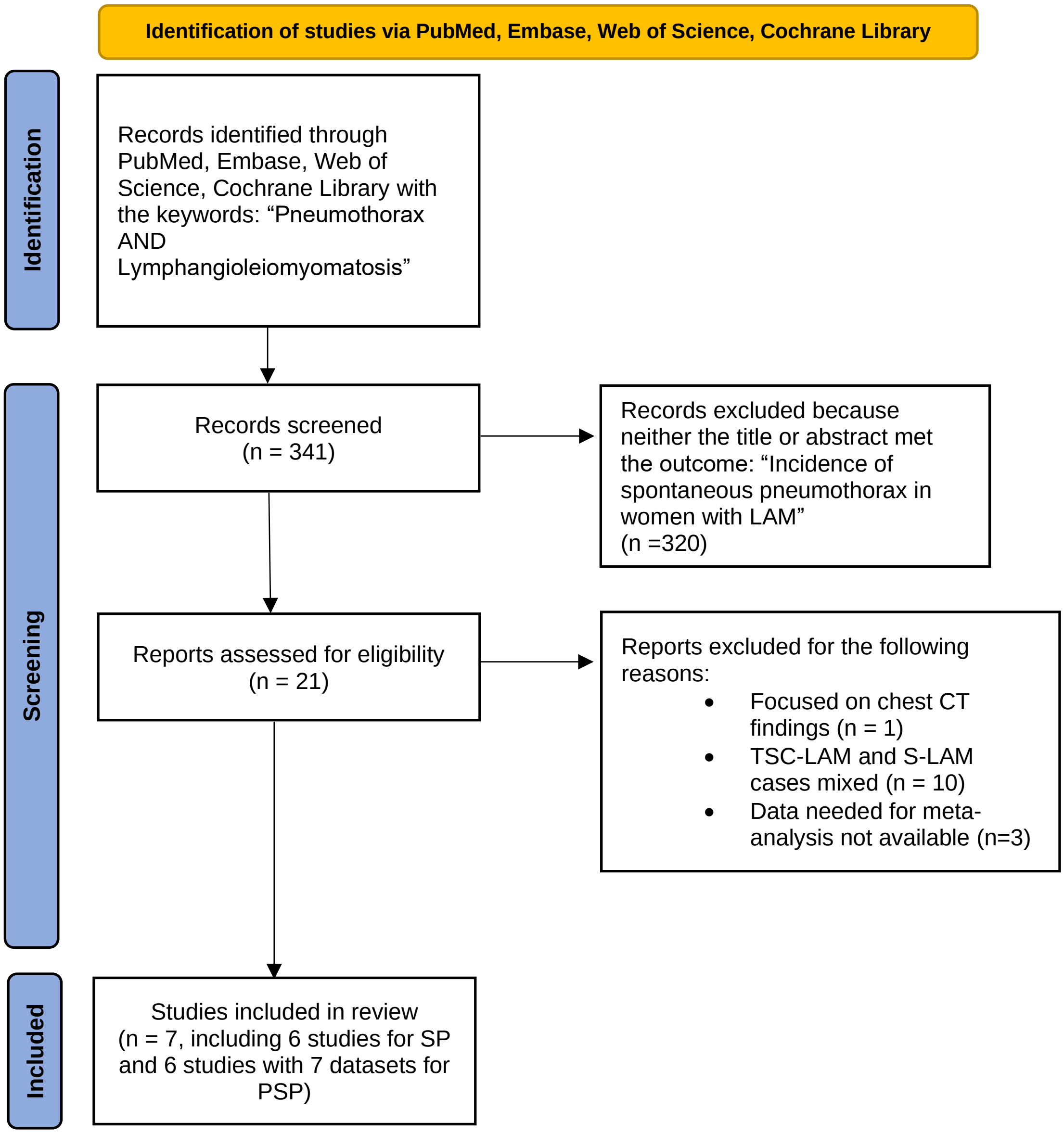

Supplementary figure S7. Prevalence of SP in women with S-LAM

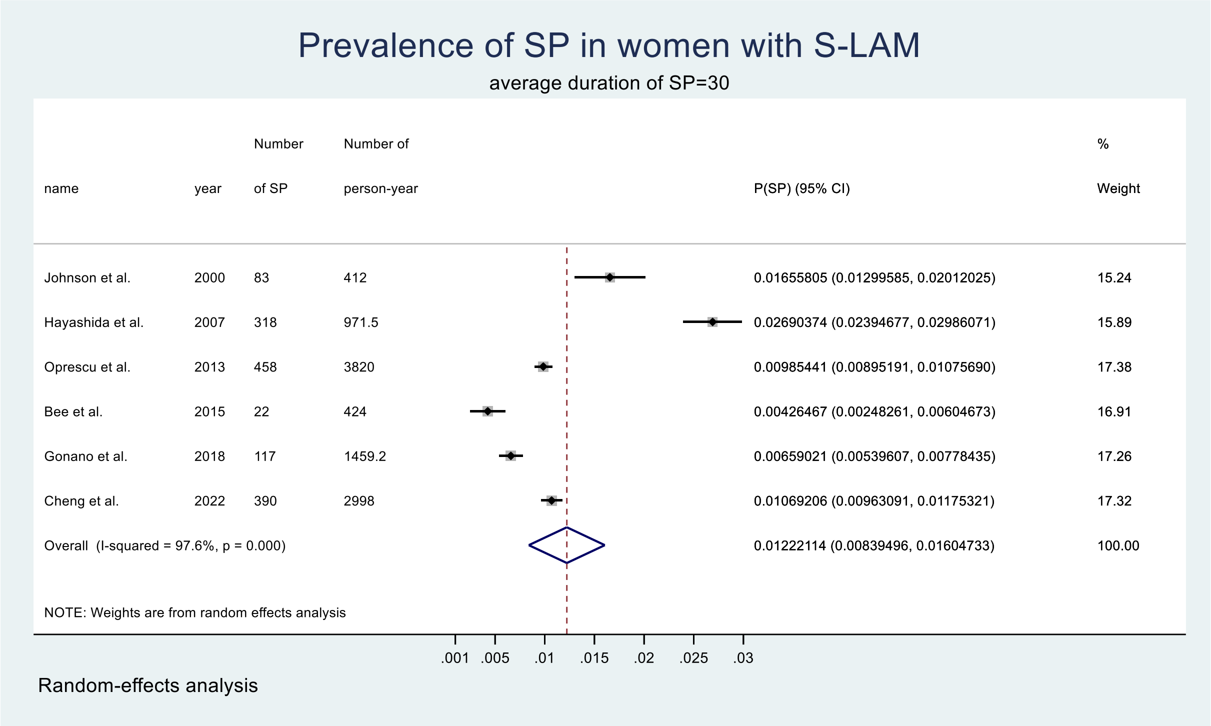

Supplementary figure S8. PRISMA flow diagram for PSP epidemiology

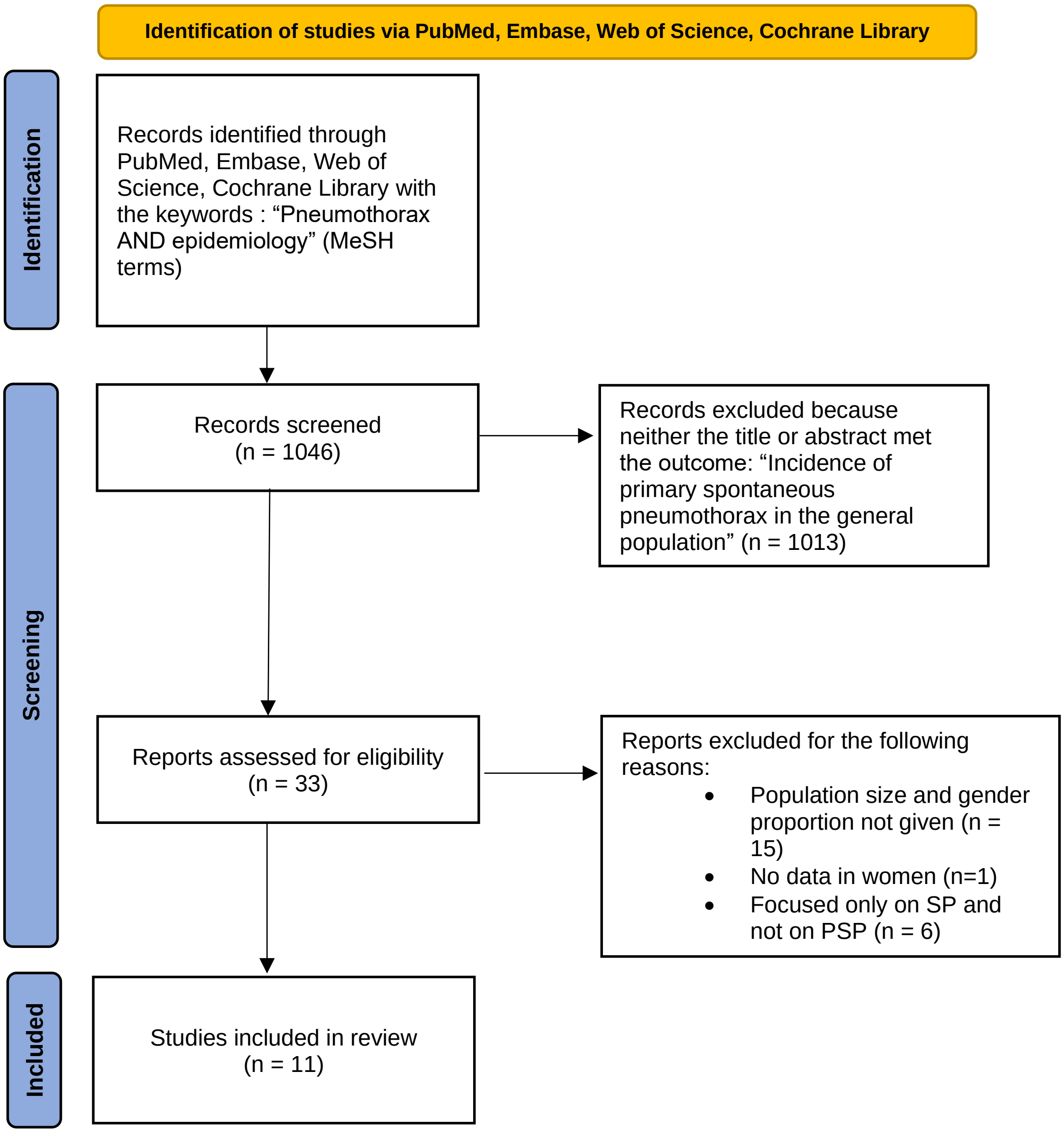

Supplementary figure S9. Incidence rate of PSP in women of the general population stratified by period before/after July 2000

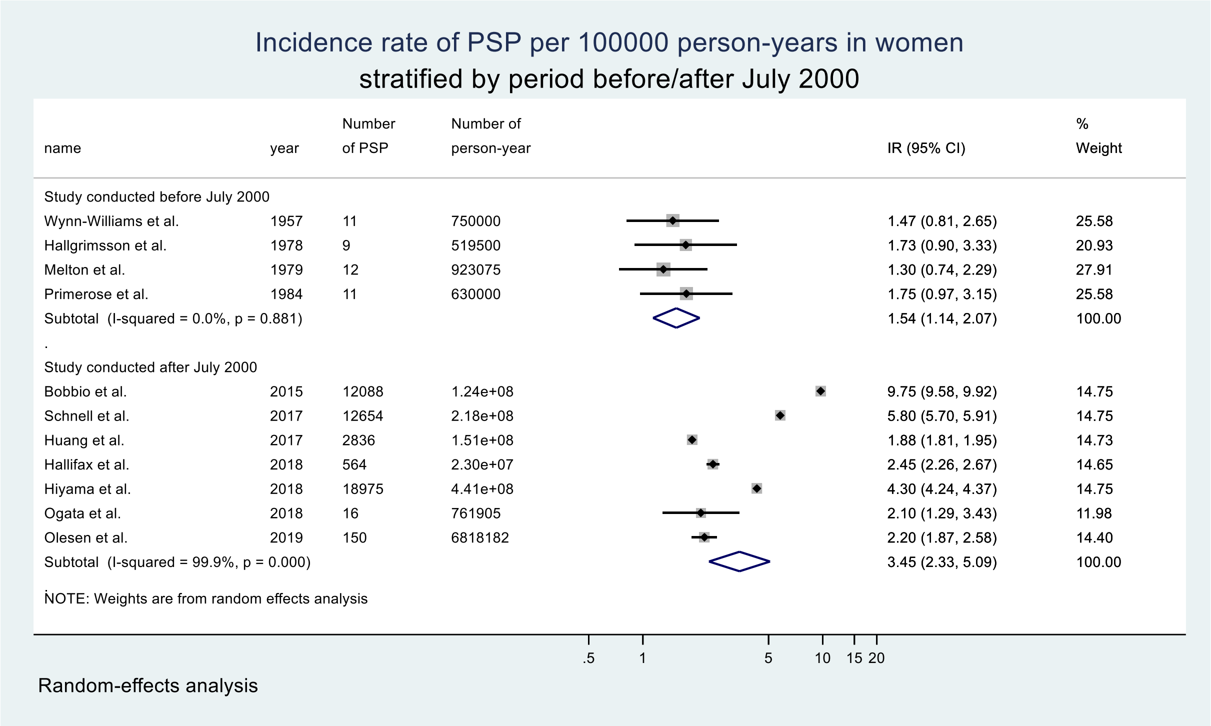

Supplementary figure S10. Overall prevalence of PSP in women of the general population, with an average PSP duration of 30 days

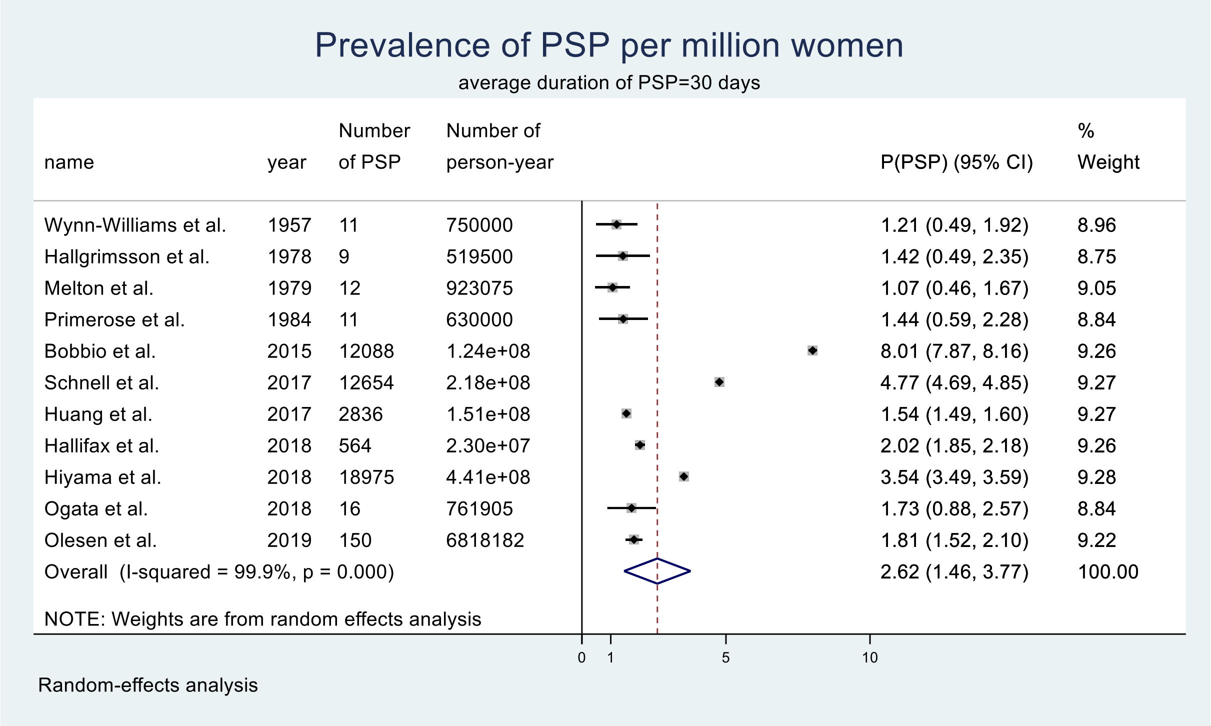

Supplementary figure S11. Prevalence of PSP in women of the general population, with an average PSP duration of 30 days, stratified by period before/after July 2000

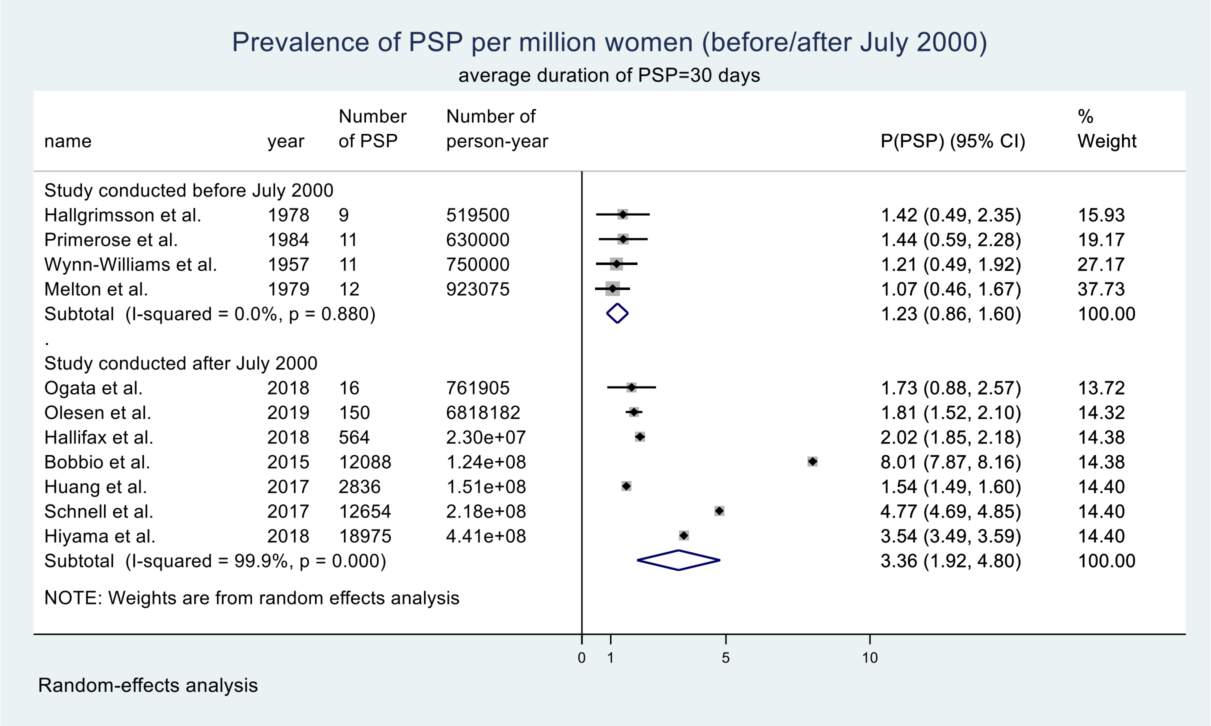

Supplementary figure S 12. Prevalence of apparent PSP in women with S-LAM, with average duration of PSP of 30 days, random-effects model.

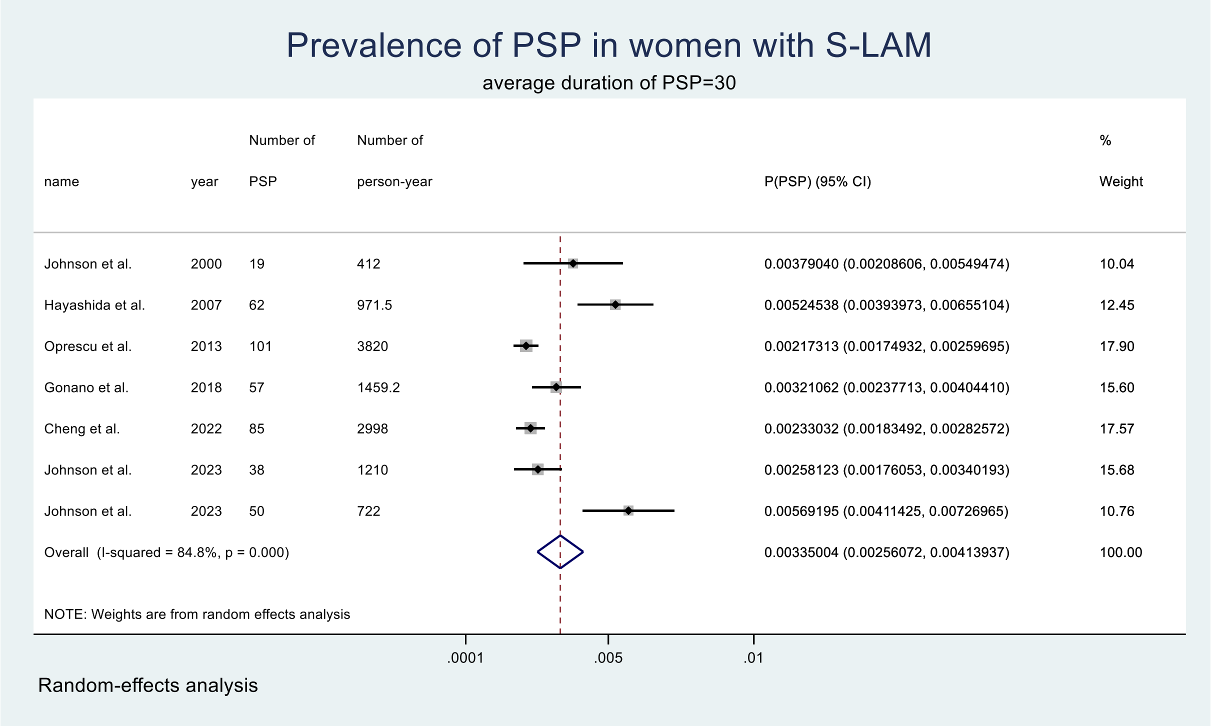

Supplement: Supplementary file 1 — Additional file 1. Fig S1. PRISMA flow diagram for S-LAM epidemiology. Fig S2. PRISMA flow diagram for SP epidemiology. Fig S3. Incidence rate of SP in women stratified by period before/after July 2000. Fig S4. Overall prevalence of SP in women with average pneumothorax duration of 30 days. Fig S5. Prevalence of SP in women stratified by period before/after July 2000, with average pneumothorax duration of 30 days. Fig S6. PRISMA flow diagram for SP and PSP in women with S-LAM. Fig S7. Prevalence of SP in women with S-LAM with average duration of SP of 30 days, random-effects model. Fig S8. PRISMA flow diagram for PSP epidemiology. Fig S9. Incidence rate of PSP in women of the general population stratified by period before/after July 2000. Fig S10. Overall prevalence of PSP in women of the general population, with an average PSP duration of 30 days. Fig S11. Prevalence of PSP in women of the general population stratified by period before/after July 2000, with average PSP duration of 30 days. Fig S12. Prevalence of apparent PSP in women with S-LAM, with average duration of PSP of 30 days, random-effects model. [file 13023_2023_2784_MOESM1_ESM.pdf]
